# Effect of a Fortified Dairy-Based Drink on Micronutrient Status, Growth, and Cognitive Development of Nigerian Toddlers- A Dose-Response Study

**DOI:** 10.3389/fnut.2022.864856

**Published:** 2022-04-27

**Authors:** Idowu Odunayo Senbanjo, Adedotun J. Owolabi, Kazeem Adeola Oshikoya, Jeske H. J. Hageman, Yetunde Adeniyi, Folake Samuel, Alida Melse-Boonstra, Anne Schaafsma

**Affiliations:** ^1^Department of Paediatrics and Child Health, Paediatric Gastroenterology, Hepatology and Nutrition Unit, Lagos State University College of Medicine, Lagos, Nigeria; ^2^Division of Human Nutrition and Health, Wageningen University and Research, Wageningen, Netherlands; ^3^Department of Pharmacology, Therapeutic and Toxicology, Lagos State University College of Medicine, Lagos, Nigeria; ^4^FrieslandCampina, Amersfoort, Netherlands; ^5^Department of Child and Adolescent Psychiatry, University College Hospital, Ibadan, Nigeria; ^6^Department of Human Nutrition, Faculty of Public Health, College of Medicine, University of Ibadan, Ibadan, Nigeria

**Keywords:** undernutrition, toddlers, Nigeria, multi-nutrient fortified dairy-based drink, growth, micronutrient status, cognition

## Abstract

Malnutrition results in a high prevalence of stunting, underweight, and micronutrient deficiencies. This study investigated the effect of a multi-nutrient fortified dairy-based drink on micronutrient status, growth, and cognitive development in malnourished [height-for-age z-score (HAZ) and/or weight-for-age z-score (WAZ) < -1 SD and >−3 SD] Nigerian toddlers (*n* = 184, 1–3 years). The product was provided in different daily amounts (200, 400, or 600 ml) for 6 months. At baseline and endline, venous blood and urine samples were collected to determine micronutrient status. Bodyweight, height, waist, and head circumference were measured, and corresponding Z-scores were calculated. The Bayley-III Screening Test was used to classify the cognitive development of the children. In a modified per-protocol (PP) population, the highest prevalence's of micronutrient deficiencies were found for vitamin A (35.5%) and selenium (17.9%). At endline, there were no significant improvements in iodine, zinc, vitamin B12, and folate status in any of the three groups. Regarding vitamin D status (25OHD), consumption of 600 and 400 ml resulted in an improved status as compared to baseline, and in a difference between the 600- and 200-ml groups. Consumption of 600 ml also increased vitamin A and selenium status as compared to baseline, but no differences were found between groups. Within the groups, WAZ, weight-for-height z-score (WHZ), and BMI-for-age z-score (BAZ) improved, but without differences between the groups. For HAZ, only the 600 ml group showed improvement within the group, but it was not different between groups. For the absolute weight, height, and head circumference only trends for differences between groups were indicated. Cognition results did not differ between the groups. Within groups, all showed a decline in the per cent of competent children for receptive language. To study the effects of a nutritional intervention on linear growth and cognition, a longer study duration might be necessary. Regarding the improvement of micronutrient status, 600 ml of fortified dairy-based drink seems most effective.

**Clinical Trial Registration:**

https://clinicaltrials.gov/ct2/show/NCT03411590?term=NCT03411590.&draw=2&rank=1, identifier: NCT03411590.

## Introduction

Undernutrition remains one of the leading causes of mortality in children under 5 years in developing countries (1, 2) and will have a negative impact on development over generations (3). Acute undernutrition, often referred to as wasting, is primarily caused by protein–energy malnutrition (PEM), whereas chronic undernutrtion is mostly the result of long-term deprivation from sufficient amounts of calories and essential nutrients and is marked by stunted vertical growth. Poor nutrition during the early developing years is associated with morbidity, mortality, growth retardation, impaired immunological functioning, and delayed mental and motor developments (4, 5). In the long term, there is an increased risk of non-communicable diseases including skeletal, cardiovascular, and metabolic disorders, as well as impaired intellectual performance and work capacity (1, 6). Annually, Nigeria loses over US$1.5 billion in Gross Domestic Product to vitamin and mineral deficiencies (7). That is why the World Health Organization (WHO) states the necessity of adequate nutrition of good quality in order to significantly reduce stunting and wasting by 2025 in the early stages of life (8). Therefore, nutrient deficiencies have to be addressed (9, 10). Micronutrients, though required in minute quantities, are important to ensure adequate growth and development. The inadequate intake of these essential nutrients can result into major health complications such as poor health, blindness, stunted growth, mental retardation, and learning disabilities (11). In Nigeria, the most common deficiencies exist for vitamin A, folate, iron, iodine, and zinc. The estimated prevalence of vitamin A deficiency especially in children aged 6 to 59 months is reported between 40 and 50% (12). Vitamin A supplementation is associated with a clinically meaningful reduction in mortality in children by about 24% (13). Other vitamins of importance are vitamin D, vitamin B12 and folic acid for their roles in growth, brain function, and immunity. Despite its low latitude, vitamin D can also be deficient (<30 nmol/L 25OHD) in Nigeria, as reported for 50% of the children from the region of Jos, and may affect bone formation, immunity and brain function (14, 15). However, in other regions vitamin D status was found to be sufficient (~ 50 nmol/L 25OHD according the WHO), or even high (~125 nmol/L) (16, 17). Vitamin B12 is essential for brain development (18), and linear growth (19). In Nigeria, about 8–36% of pregnant women from Jos, and 9% adolescent girls in Northern Nigeria were reported to be vitamin B12 deficient (20, 21). Folic acid is recognised as crucial for nervous system development and maintenance, and brain maturation (22, 23). Although folic acid deficiency was low in adolescent girls from Maiduguri in Nigeria (23), 60% of children in Ibadan had an inadequate dietary intake of folate (24).

With regard to minerals, zinc status is associated with incidence, severity, and duration of childhood diarrhoea, as well as with growth (25–27). In Nigeria, zinc deficiency is reported in 20–99% of children aged <5 years, depending on the region (28–30). As iodine deficiency is well-known, fortification programmes (in particular salt iodisation) are in place in many African countries (31). Iodine plays an important role in thyroid function, and as such also in brain development and function (32). Based on food intake, 1–10% of the population of Nigeria has a risk of low iodine status (31). When talking about iodine, an adequate selenium status is necessary to control hydrogen peroxide that is released in the production of thyroid hormones (33). On average, selenium deficiency is estimated to be 0–10% in the Nigerian population (31).

In a systematic review, a positive, although non-significant, impact of multi-micronutrient fortification on height-for-age (HAZ), weight-for-age (WAZ), and weight-for-height Z-scores (WHZ) was reported (34). However, energy-rich lipid-based nutrient supplements (LNS), providing lipids, essential fatty acids, protein, and micronutrients, might be more effective in improving growth in malnourished children (35–37). With regard to protein, intake appears to be adequate in Nigerian children, though, the quality of protein in developing countries may be questioned (38). Additional high-quality protein could support neurodevelopment and catch-up growth in stunted or wasted children (39–41).

Based on the above, a multi-micronutrient fortified food or beverage containing micronutrients, fat, carbohydrates, and high-quality protein is potentially a good option to support the growth and development of children. The affordability of such fortified products is still a matter of concern; hence it is necessary to assess daily effective volumes of intake. We reported earlier on the efficacy of different volumes of a multi-micronutrient fortified dairy-based drink on anaemia and gut microbiota in malnourished Nigerian toddlers aged 12–36 months (42). In this paper, we report on the efficacy of the drink on the biochemical status of other micronutrients, as well as possible effects on growth and cognition.

## Materials and Methods

### Subjects and Study Design

In this three-arm, open (blind for biochemical analyses) randomised intervention trial, apparently healthy Nigerian toddlers (1–3 years) (*n* = 184) with mild-moderate anaemia (Hb ≥ 7.0 and ≤ 10.9 g/dL) and mild-moderate malnutrition (HAZ and/or WAZ < -1 SD and >-3 SD), were recruited in Ijora-Badia community in Apapa-Iganmu Local Council Development Area (LCDA), Lagos, South-West Nigeria. Children with severe malnutrition and anaemia were excluded since they require additional measures such as hospital admission and blood transfusion. All subjects were able to consume a maximum of 600 mL of product per day at the time of inclusion. Children were not included when they (I) had a chronic or severe illness requiring hospitalisation and/or special treatment, (II) had a recent medical history (past 3 months) of serious infections, injuries and/or surgeries, (III) had any known allergies or intolerances to milk or milk ingredients, (IV) were predominantly breastfed toddlers, (V) consumed any other fortified foods or supplements, (VI) participated in micronutrient supplementation programs, (VII) participated in any other nutritional study in the last 6 months, (VIII) were likely to move within the period of intervention, (IX) when parents or guardians were related to or employed by the sponsor or the university, (X) used any prescription medications before and/or during the study period for ≥2 weeks. No restrictions were set for regular food intake. Families with toddlers that were permanent residents of Ijora-Badia were informed about the study by a mobilisation team from the State Ministry of Health working in the Ijora Badia community a few weeks before the commencement of the study. During information meetings, parents, or legal guardians of potential candidates (toddlers) received detailed information about the study, the requirements, and procedures, and all their questions were answered. At screening, after a signed informed consent was obtained from parents or legal guardians, trained researchers verified age (by birth certificate confirmation or caregiver) and took anthropometric measurements. Eligible children were directly enrolled and randomly assigned to one of the three study groups by the principal investigator. For randomisation, a computer-generated block-randomisation, based on the order of screening and stratified for gender and age (12–27, and 28–36 months of age) was used, with an allocation ratio of 1:1:1. Following inclusion, study participants received deworming treatment (10 mg/kg bodyweight pyrantel pamoate), to ensure that expected worm infections would not interfere with the study treatment. Afterwards, baseline measurements were performed. Venous blood samples (10 mL) were taken for the assessment of nutritional status parameters. Samples of early morning urine were collected before the start of the intervention. All measurements were repeated at the end of the 6-month intervention period. WHO Anthro 2007 (43) was used to generate WAZ, HAZ, WHZ, and BMI-for-age (BAZ) scores.

### Ethics

The study was conducted in accordance with the Declaration of Helsinki, and the protocol was approved by the Ethics Committee of Lagos State University Teaching Hospital (LREC/10/06/829). The trial was registered at ClinicalTrials.gov: NCT03411590.

### Study Products

The three groups received a multi-nutrient fortified dairy-based drink (Peak 123, FrieslandCampina WAMCO, Lagos, Nigeria), in amounts of 200, 400 or 600 mL per day. In the case of 400 and 600 mL, parents were requested to spread the portions (200 mL each) over the day. The time of intake was not monitored. The composition of the drink is shown in Table 1. The ingredient list is presented in Supplementary Table 1. Airtight packed powder sachets (for 200 mL each) were delivered weekly to the families by trained researchers who also provided instructions for use. Consumption of test products started as soon as all baseline examinations were completed and baseline blood, early morning urine, and faecal samples were collected. In the case of twins and siblings, only the child who met the inclusion criteria was enrolled in the study, however, the other child received the same treatment to prevent sharing and non-compliance with the study protocol.

**Table 1:** Composition of the multi-nutrient fortified dairy-based drink, provided to Nigerian toddlers with malnutrition in daily amounts of 200, 400 or 600 ml, during 6 months.

**Nutrient**	**Unit**	**Per 200 ml**	**Per 400 ml**	**Per 600 ml**
Energy	kcal	149	297	446
Protein	g	5	11	16
Carbohydrates	g	20	41	61
Sucrose	g	13	27	40
Fat	g	5	10	15
DHA	mg	14	28	42
Calcium	mg	188	376	564
Phosphorus	mg	152	304	455
Potassium	mg	244	488	733
Magnesium	mg	17	33	50
Sodium	mg	63	125	188
Iron	mg	2.24	4.48	6.72
Copper	ug	58	116	173
Zinc	mg	1	2	3
Iodine	ug	40	79	119
Selenium	ug	3.6	7.3	11
Vitamin A	ug-RE	128	255	383
Vitamin D3	ug	2	4	6
Vitamin E	mg	3	5	8
Vitamin B1	ug	155	310	465
Vitamin B2	ug	158	317	475
Vitamin B6	ug	157	314	470
Folic acid	ug	24	48	71
Vitamin B12	ug	0.4	0.8	1,2
Vitamin K1	ug	9.2	18.5	28
Biotin	ug	5.3	10.6	16
Niacin	mg	2.0	4.0	6
Pantothenic acid	mg	0.7	1.3	2
Vitamin C	mg	38	76	114

### Micronutrient Status Parameters in Blood

Venous blood sampling (10 ml in total) was performed in the morning between 9:00 and 11:00 a.m. following an overnight fast of at least 12 hrs. The blood was collected in an EDTA microtainer (4 ml), heparin gel microtainer (4 ml), and a serum microtainer (2 ml). The EDTA and heparin microtainers were kept at 4°C and transferred (on ice) to a local laboratory on the day of collection. In the laboratory, tubes were directly centrifuged (HaematoSpin 1400, Hawksley, UK) at 3,300 g for 15 min and the extracted EDTA and heparin plasma was pipetted into aliquots of 200 μL. Serum microtainers were kept at room temperature for at least 60 min to allow clotting. Clotted blood was centrifuged at 2,000 g for at least 3 min and the extracted serum was pipetted into aliquots of 200 μL. All serum and plasma aliquots were stored at −20°C and transported on dry ice to the Amsterdam University Medical Center (Location Vumc, Amsterdam, The Netherlands), and Medlon B.V. (Enschede, The Netherlands), for biochemical analyses.

EDTA plasma was used for the analysis of zinc, selenium and 25-hydroxyvitamin D (25OHD_3_ and 25OHD_2_). For zinc and selenium analyses, samples were diluted 30 × using 0.2% v/v HNO3 0.05% v/v Triton 1% v/v Methanol and analysed by inductively coupled plasma mass spectrometry (ICP-MS) using a kinetic energy discrimination procedure on the Perkin Elmer Nexion 300× ICP-MS. Cut-off values used for zinc and selenium sufficiency were 10 μmol/l (44) and 0.8 μmol/L (45). Vitamin D was analysed using an optimised LC-MS/MS method, as described by Dirks et al. (46) (referring to method E). For this study 2, cut-off values for 25OHD are considered: 50 and 75 nmol/L; 50 nmol to be sufficient for skeletal metabolism and 75 nmol for extra-skeletal activities (14, 47, 48).

Heparin plasma was used for the analysis of folate and vitamin A. Folate was analysed using the Elecsys Folate III binding assay and Cobas-e immunoassay analyser (Roche/Hitachi); measuring range: 0.6–20.0 or 1.36–45.4 nmol/L. The cut-off value for folate sufficiency used in this study was 10 nmol/L (45). Vitamin A was determined using isocratic high-performance liquid chromatography with UV detection. Intra-assay CV was 0.6–0.9%, whereas the inter-assay CV was 1.0–1.3%, with a lowest detection limit of 0.1 μM (49). The cut-off value for vitamin A sufficiency was 0.7 μmol/L (50).

Vitamin B12 was determined in serum using the Elecsys Vitamin B12 binding assay and Cobas-e immunoassay analyser. Measuring range: 50.0–2,000 pg/ml or 36.9–1,476 pmol/L. The cut-off value for vitamin B12 sufficiency used in this study was 150 pmol/L (45).

### Urinary Iodine Analysis

The iodine status of the children was assessed by measuring the iodine concentration in early morning spot urinary samples (5 ml) before any food or drink was consumed, collected into a 10-ml universal laboratory bottle, at baseline and after the intervention. The samples were brought to the collection centres by parents and/or caregivers within 1 h, stored at 5–7°C, and transferred on ice to the nearest freezer (-20°C) at the end of the day. For analysis, frozen samples were transported on dry ice to the Central Clinical Laboratory of the University Medical Center Groningen, the Netherlands. Iodine in urine was analysed using ICP-MS (Varian, Varian Inc., Palo Alto, USA; the lowest level of quantification (LLOQ) 25 μg/L). The cut-off value used for iodine deficiency in the study population sample was a median value of <100 ug/L, whereas ≥300 μg/L was used as a cut-off for iodine excess (51).

### Anthropometry

Bodyweight, recumbent length or standing height (for toddlers up to 24 months old or 24 to 36 months respectively), head circumference (HC), and mid-upper arm circumference (MUAC) were measured in triplicate at baseline (screening) and after 6 months (at home). All measurements were taken and recorded by well-trained team members. For bodyweight, a SECA electronic scale (Seca gmbh & co., Hamburg, Germany) appropriate for infants and toddlers was used having a precision of ±20 g for weights below 20 kg and ±50 g for weights up to 50 kg. The scale was placed on a flat, stable surface and every effort was made to ensure that restless toddlers were calm during the weighing procedure. When the child was not calm enough, the child was weighed together with the parent or caregiver. The weighing scale was calibrated daily using a weight standard of 10 kg. All children were weighed undressed, without a diaper, jewellery, or other ornaments. Recumbent length (children 12–24 months of age) was measured to the nearest 0.1 cm using Seca 417 Light and stable measuring board (Seca gmbh & co, Hamburg, Germany) with a stationary headpiece, a sliding vertical foot piece and a horizontal back piece with a measuring tape mounted on it. All children were measured without shoes or any other footwear. Any haircut influencing length was considered. The measuring board was calibrated daily using a length standard of 40 cm.

In 24–36-month-old toddlers, standing height was measured to the nearest 0.1 cm using Seca 213 Mobile stadiometer for measuring height (Seca gmbh & co, Hamburg, Germany). The measurement of height was conducted without shoes and with children keeping their shoulders in a relaxed position, their arms hanging freely, and their heads aligned in Frankfurt plane. Any haircut influencing length was considered. The stadiometer was calibrated daily using a length standard of 80 cm.

Weight and length were used to calculate Body Mass Index (BMI; kg/m^2^). WHO Anthro 2007 (52) was used to generate WAZ, HAZ, WHZ, and BAZ scores.

HC and MUAC were measured to the nearest 0.1 cm with the use of a flexible, non-stretchable, measurement tape (Lufkin W606PM tape, Hoechstmass Ballzer GmbH, Sulzbach (Taunus), Hessen, Germany) and with the toddler at a sitting (HC) and standing position (MUAC). HC was measured after aligning the head in Frankfurt plane and passing the measuring tape around the head, just above the eyebrows, above the ears on each side and over the occipital prominence at the back of the head to its maximal circumference. MUAC of the left upper arm was measured at the mid-point between the tip of the shoulder and the tip of the elbow (acromion and the olecranon process respectively). MUAC is a precise, sensitive and accurate method and parameter for the identification of undernutrition among children aged under 5 years (53).

### Cognitive Development

Cognitive assessment of children was done using the short (screening) version of the Bayley-III (BSID, Bayley Scales of Infant and Toddler Development®, Third Edition, Bayley–III) (54). The BSID is an internationally, a multi-scale neuro-developmental battery designed for use in infants and young children from 0 to 42 months (54). The test has been used in some African cultures and is said to be a valid developmental assessment scale for Nigerian children (55). Items in the subtest are particularly valuable in quick screening high-risk infants for developmental delay with regard to five domains, namely expressive and receptive language, cognition, and fine and gross motor areas (54). The test results in a score per development area, and based on cut-off scores, as provided by the BSID, the children can be classified as At Risk, Emerging, or Competent for each cognitive area (Supplementary Table 2). Good reliability coefficients (>0.9) have been established for all the Bayley-III tests, both in the general population and special groups. The BSID takes 15–25 min to complete (54).

The test was carried out by two trained licenced neuro-developmental psychologists. These experts attended a 3-day refresher course on how to administer the BSID, including a 2-day practical exposure in the field. The mean interrater reliability of the test between the trainer and the psychologist was 0.98, while the interrater reliability between the two psychologists was 0.97. All tests took place within the study Ijora-Badia community at both baseline and endline on a one-on-one basis. The test was administered to children in the presence of their mothers, or caregivers in a quiet room that was well-lit, well-ventilated, and free from distractions according to the standard of the testing of Bayley. The parents were asked to sit beside their toddlers as it helped the child to concentrate. The test used a set of standardised toys and a detailed scoring sheet to provide a quick assessment of the five key developmental domains.

### Sample Size Calculation

The initial sample size calculation, based on a reduction in anaemia has been described previously (42). To indicate that this sample size would also be suitable for other micronutrients, the calculation was repeated for vitamin D. Depending on the region in Nigeria, the serum level of 25OHD in children differs considerably. In this calculation we used an average level of 51.2 ± 15.5 nmol/L 25OHD (17). Furthermore, based on the provided amount of vitamin D, the increase in 25OHD can be estimated by using a response factor of 1.2 or 2 nmol/L per μg of daily oral vitamin D during 5 months of supplementation (56). A high response factor will be likely in malnourished children. Using a power of 0.8 and α of 0.05, and for finding a significant difference with baseline, the number of children to be included in the 600 ml group was 47 or 26 (for response factors 1.2 and 2 respectively). With a dropout of 30% these numbers increased to 61 and 32.

### Statistical Analysis

**Figure 1:**
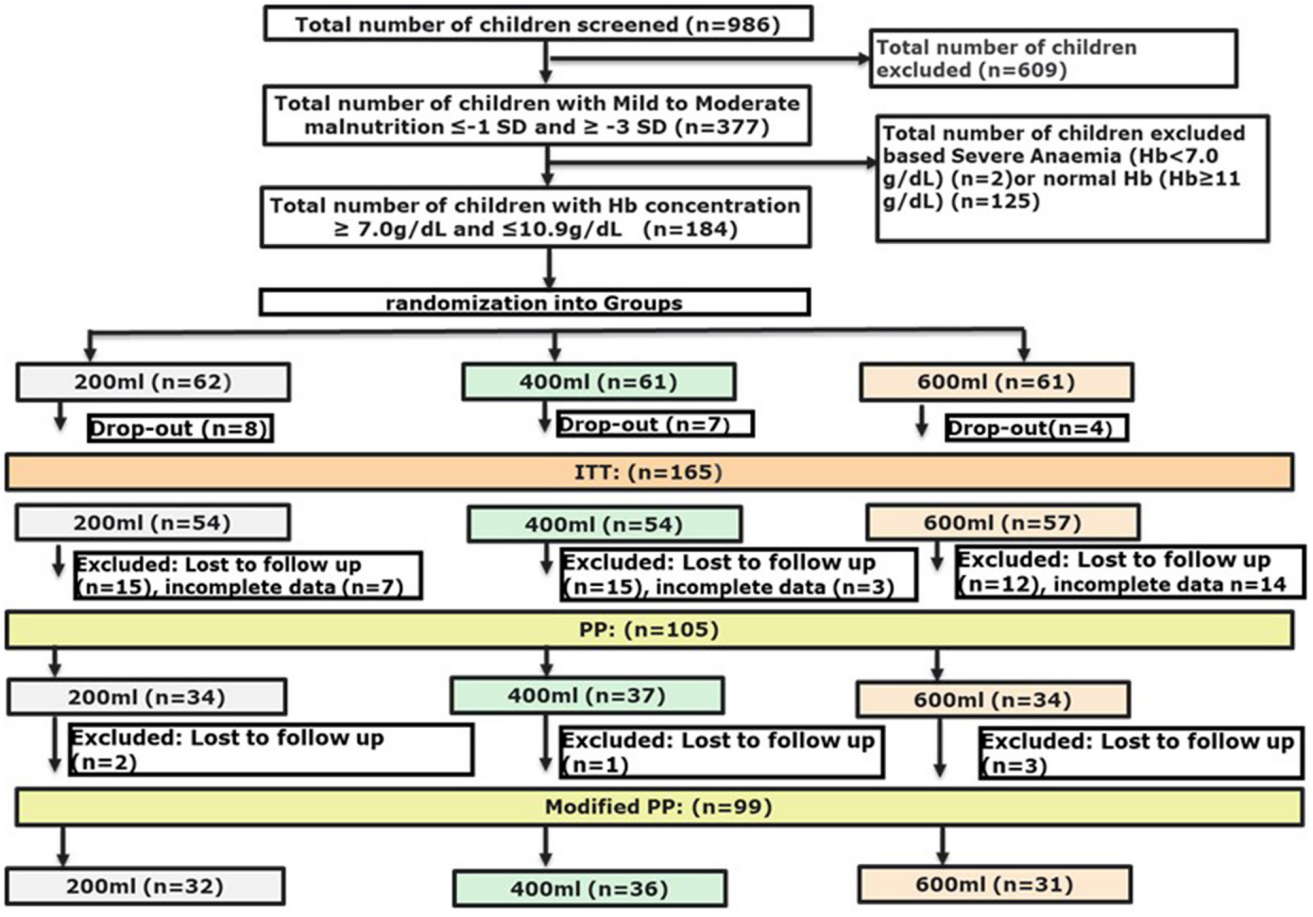
Flow-chart of screening and randomisation process.

We used a modified PP population with at least 50% of the cases with micronutrient data at endline, and with weight and height measurements available at both base- and endline, which resulted in a population that was slightly smaller (105 vs. 99 subjects) as compared to the initial PP population described earlier (42) (Figure 1). ANCOVA was used to determine the differences in effect between the interventions on the micronutrients measured. *Post-hoc* analyses were performed with a Bonferroni adjustment. Although not all micronutrients were normally distributed, ANCOVA was considered robust enough to be applied for these micronutrients as well. The change in micronutrient status from baseline to endline within intervention groups was tested with either a paired *t*-test or a related-samples Wilcoxon signed-rank test per study group. Differences in growth outcomes between intervention groups were compared using Generalised Linear Models with the study arm as a factor, and the corresponding baseline outcome as a covariate. To determine changes from baseline to endline within intervention groups, growth outcomes were tested with a paired *t*-test. The main outcomes of the Bayley-III Screening Test were the absolute numbers and the percentages of participants in each of the 3 subtest classifications: at risk, emerging or competent, for each of the five domains. From the absolute scores of the subtests, delta-values were calculated to study the effect of the intervention period. Differences in delta-values were compared between groups with one-way ANOVA. Changes in the percentages from baseline to endline within each intervention group and differences between intervention groups at endline were tested with a Fisher's Exact test.

A *p*-value < 0.05 was considered significant, whereas *p*-values < 0.1 were considered trends. All analyses were performed using IBM SPSS Statistics version 24 (IBM Corp, Armonk, NY, USA).

## Results

Baseline characteristics of the three groups within the modified PP population are presented in Table 2. No significant differences were found between the three groups, except for height and a borderline difference for HAZ, which were lower in the 600 ml group compared to the 200 ml group (*p* = 0.016 and *p* = 0.05, respectively). An overview of baseline characteristics of the ITT population can be found in Supplementary Table 3.

**Table 2:** Baseline characteristics of the modified PP population, a study in which Nigerian toddlers with malnutrition were provided with a fortified dairy-based drink in daily amounts of 200, 400 or 600 ml, during 6 months (presented as mean ± SD or median, IQR).

	**200 ml**	**400 ml**	**600 ml**	***p*** **-value**
**N**	**32**	**36**	**31**	
Age (months)	21.0, 9	20.0, 8	18.0, 8	0.281°
Sex (% male/female)	50.0/50.0	52.8/47.2	38.7/61.3	0.486^#^
Religion (% Christian/Muslim)	29.0/71.0	34.3/65.7	29.0/71.0	0.865^#^
Family setting (% Monogamy/Polygamy)	79.3/20.7	73.5/26.5	75.0/25.0	0.861^#^
Social class (upper/middle/lower/lowest)	0/22.6/77.4/0	2.9/20.0/71.4/5.7	0/16.1/77.4/6.5	0.661^#^
Weight (kg)	9.3, 2.5	8.9, 1.1	8.6, 1.7	0.238°
Height (cm)	79.2 ± 5.4^a^	77.9 ± 4.6^a, b^	75.7 ± 4.3^b^	0.016*
BMI (kg/m^2^)	14.6 ± 0.9	14.9 ± 0.9	15.0 ± 1.1	0.212*
Head circumference (cm)	46.7 ± 1.8	46.6 ± 1.3	46.5 ± 1.5	0.858*
Mid-upper arm circumference (cm)	13.7 ± 1.1	13.8 ± 0.7	13.8 ± 0.9	0.843*
Weight-for age z-score (WAZ)	−1.8 ± 0.6	−1.7 ± 0.6	−1.8 ± 0.5	0.842*
Height-for-age z-score (HAZ)	−1.6 ± 0.6	−1.8 ± 0.5	−2.0 ± 0.6	0.050*
Weight-for height z-score (WHZ)	−1.3 ± 0.8	−1.2 ± 0.7	−1.1 ± 0.7	0.428*
BMI-for-age z-score (BAZ)	−1.1 ± 0.8	−0.9 ± 0.7	−0.8 ± 0.8	0.291*
Iodine (ug/L)	252.6, 402.0	323.0, 426.5	315.8, 492.1	0.968°
Zinc (mmol/L)	12.1, 4.0	11.0, 3.1	11.8, 3.0	0.076°
Selenium (μmol/L)	1.0, 0.2	0.9, 0.2	0.9, 0.2	0.314°
Vitamin A (umol/L)	0.9 ± 0.3	0.8 ± 0.3	0.7 ± 0.2	0.134*
Vitamin B12 (pmol/L)	620.0 ± 291.6	560.9 ± 269.0	618.1 ± 281.4	0.704*
Folate (nmol/L)	19.7 ± 7.2	21.7 ± 7.4	21.5 ± 13.0	0.663*
Vitamin D3 (nmol/L)	68.0, 20	65.0, 21	67.0, 24	0.850°

*Output of °Kruskal-Wallis test*,

# Chi-square test, or * one-way ANOVA. Different letters in superscripts ^a, b^ indicate differences between intervention groups.

### Micronutrients

Micronutrient status was determined in blood and urine before and after the intervention. Table 3 shows no differences at endline between the three interventions for iodine, zinc, selenium, vitamin B12, folate, and vitamin A. Only vitamin D3 was higher in the 600 ml group as compared to the 200 ml group (*p* = 0.012). C-reactive protein concentrations, as an indicator of acute inflammation, were reported previously and considered to be normal (<5 mg/L) for the majority of the study participants (42).

**Table 3:** Micronutrient status at the endline of the modified PP population, a study in which Nigerian toddlers with malnutrition were provided with a fortified dairy-based drink in daily amounts of 200, 400 or 600 ml, for 6 months (data are presented as mean ± SD or median, IQR).

	**200 ml** **(***n*** = 32)**	**400 ml** **(***n*** = 36)**	**600 ml** **(***n*** = 31)**	***p*** **-value^**1**^**
Iodine (ug/L)	377.0, 331.0	394.5, 344.5	337.0, 544.5	0.782
Zinc (μmol/L)	11.4 ± 1.7	11.2 ± 1.9	11.4 ± 2.4	0.922
Selenium (μmol/L)	0.9, 0.2	1.0, 0.1	1.0, 0.2	0.695
Vitamin A (μmol/L)	0.8, 0.3	0.8, 0.2	0.9, 0.4	0.093
Vitamin B12 (pmol/L)	521.0, 229	495.0, 317	463.5, 198	0.734
Folate (nmol/L)	19.3 ± 6.3	18.9 ± 7.5	17.1 ± 6.4	0.449
Vitamin D3 (nmol/L)	73.0, 15^a^	79.0, 21^a, b^	80.0, 21^b^	**0.012**

*^1^The p-value represents the outcome of the ANCOVA, controlling for baseline values*.

*Superscripts ^a, b^ indicate differences between intervention groups*.

**Figure 2:**
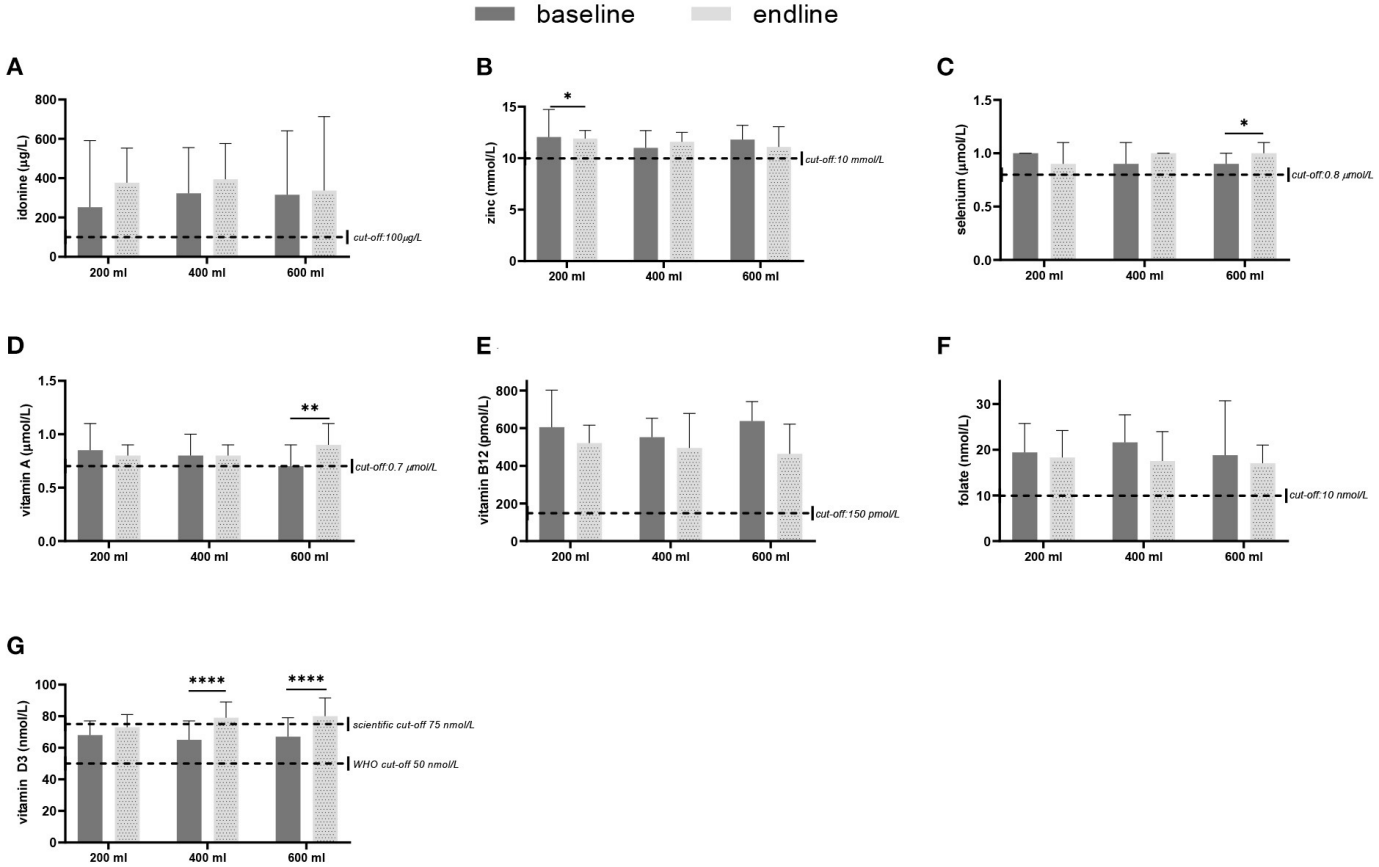
Micronutrient levels in blood or urine, sampled at baseline and after 6 months of intervention with a fortified dairy-based drink in daily amounts of 200, 400 or 600 ml, in Nigerian toddlers with malnutrition: **(A)** Iodine (median + IQR), **(B)** zinc (mean + SD), **(C)** selenium (median + IQR), **(D)** vitamin A (median + IQR), **(E)** vitamin B12 (median + IQR), **(F)** folate (mean + SD), and **(G)** vitamin D3 (median + IQR) for the three study groups at baseline and after the 6 months intervention. The difference between baseline and endline was tested with either a paired *t*-test or a related-samples Wilcoxon signed-rank test per study group. *p < 0.05, **p < 0.01, ****p < 0.0001.

In Figure 2 the base- and endline values of the micronutrients and the outcomes of the within-group comparisons are shown. A small decrease in zinc concentration was found after the intervention with 200 ml compared to baseline (*p* = 0.047). Consumption of the multi-nutrient fortified formula increased vitamin D3 status (*p* < 0.0001) in both the 400- and 600-ml group, but not in the 200 ml group. The intervention with 600 ml of multi-nutrient fortified formula per day also increased selenium (*p* = 0.022) and vitamin A (*p* = 0.003) levels. Table 4 shows the prevalence of deficiencies or sub-normal levels for the different micronutrients per intervention group at baseline and after 6 months of intervention. The median urinary iodine excretion was 309 μg/L (range: 22–5622) at baseline indicating a population with an excessive iodine intake. A minority of 13.2% of the subjects had an iodine excretion of <100 μg/L (low iodine status), which decreased to 10.9% after the intervention. Zinc deficiency increased during the study from 17.2% at the start to 26.0% at the end of the study. Of the total group, 17.9% was selenium deficient before starting the intervention, and this decreased to 5.3%. More than one-third of the study subjects (35.5%) suffered from vitamin A deficiency, this decreased to 22.3%. Vitamin B12 deficiency was only present in 2.9% of the study population before the intervention, and this hardly changed during the study (1.2% at endline). A deficiency of folate was found in 9.7% of the subjects, which decreased to 6.1%. Vitamin D sub-normal status was present in 16.7% of the study population (using the WHO cut-off value of 50 nmol/L 25OHD), which decreased to 3.1% after the intervention.

**Table 4:** Prevalence (%) of sub-normal base- and end-line micronutrient status in the modified PP population of malnourished Nigerian toddlers who received a fortified dairy-based drink in daily amounts of 200, 400 or 600 ml, during 6 months.

	Reference value*	**200 ml**		**400 ml**		**600 ml**	
		**Baseline**	**Endline**	**Baseline**	**Endline**	**Baseline**	**Endline**
Iodine	100 ug/L	*n* = 28 7.1 (2.0–22.6)	*n* = 29 10.3 (3.6–26.4)	*n* = 33 15.1 (6.7–30.0)	*n* = 34 8.8 (3.0–23.0)	*n* = 30 16.7 (7.3–33.6)	*n* = 29 13.8 (5.5–30.6)
Zinc	10 μmol/L	*n* = 32 6.3 (1.7–20.1)	*n* = 31 25.8 (13.7–43.2)	*n* = 36 25.0 (13.8–41.1)	*n* = 35 28.6 (13.6–45.1)	*n* = 31 19.4 (9.2–36.3)	*n* = 30 16.7 (7.3–33.6)
Selenium	0.8 μmol/L	*n* = 32 18.8 (8.9–35.3)	*n* = 30 6.7 (1.8–21.3)	*n* = 32 12.5 (5.0–28.1)	*n* = 35 2.9 (0.5–14.5)	*n* = 31 22.6 (11.4–39.8)	*n* = 31 6.5 (1.8–20.7)
Vitamin A	0.7 μmol/L	*n* = 29 34.5 (19.9–52.7)	*n* = 30 26.7 (14.2–44.4)	*n* = 34 32.4 (19.1–49.2)	*n* = 34 23.5 (12.4–40.0)	*n* = 30 40.0 (24.6–57.7)	*n* = 30 16.7 (7.3–33.6)
Vitamin B12	150 pmol/L	*n* = 23 0.0 (0.0–14.3)	*n* = 27 0.0 (0.0–12.5)	*n* = 27 7.4 (2.1–23.4)	*n* = 31 3.2 (0.6–16.2)	*n* = 18 0.0 (0.0–17.6)	*n* = 28 0.0 (0.0–12.1)
Folate	10 nmol/L	*n* = 20 5.0 (0.0–16.1)	*n* = 27 3.7 (0.7–18.3)	*n* = 25 8.0 (2.2–25.0)	*n* = 29 6.9 (1.9–22.0)	*n* = 17 23.5 (9.6–47.3)	*n* = 26 7.7 (2.1–24.1)
Vitamin D	50 nmol/L	*n* = 29 13.8 (5.5–30.6)	*n* = 32 3.1 (0.6–15.7)	*n* = 36 16.7 (7.9–31.9)	*n* = 35 0.0 (0.0–9.9)	*n* = 31 19.35 (9.2–36.3)	*n* = 30 3.3 (0.6–16.7)

* *Reference value for zinc, selenium, vitamin B12, folate and vitamin D3 (45), iodine (51), vitamin A (50). Data are reported as: number of children included (n = x), % of children below the reference value, and the 95% confidential interval (x-x)*.

### Anthropometry

**Figure 3:**
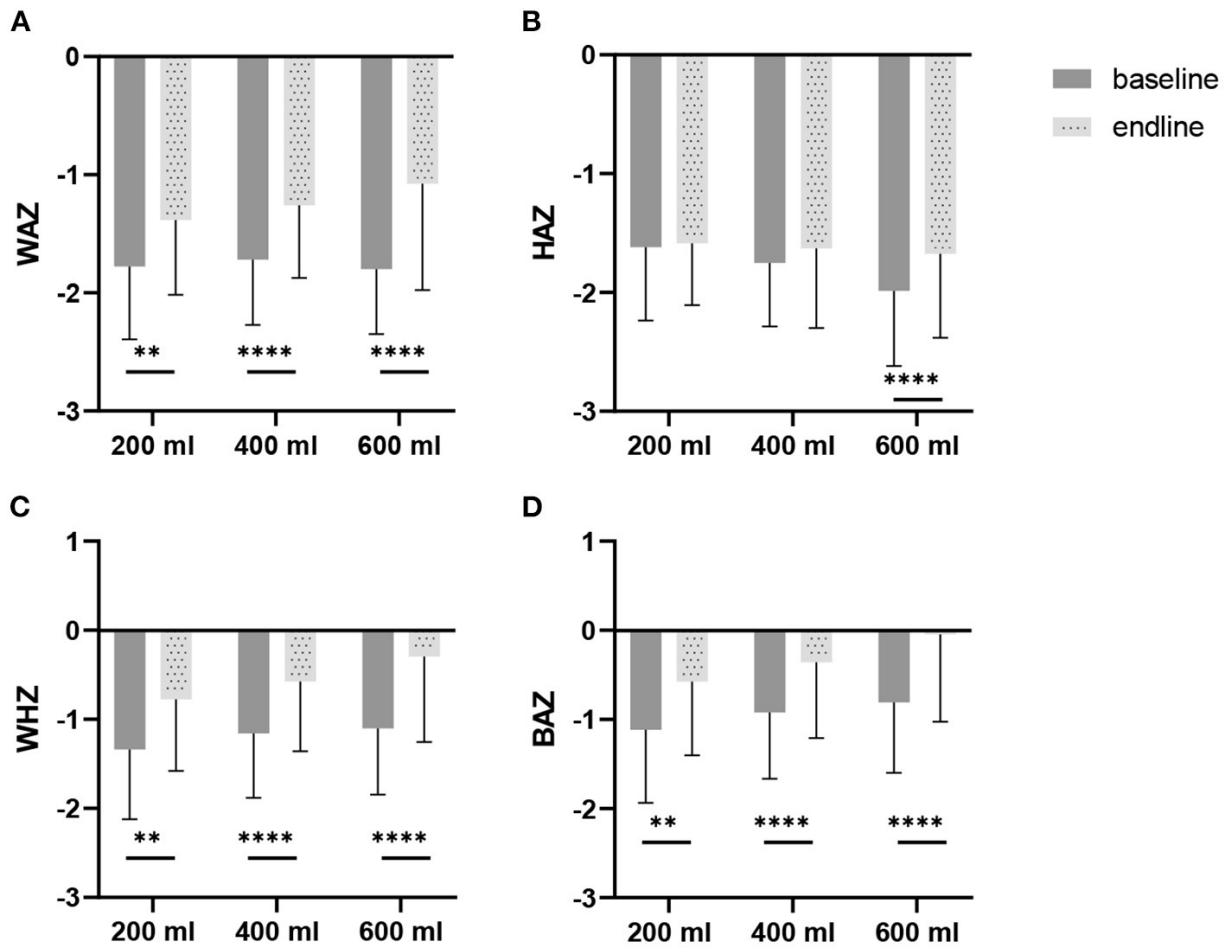
Weight-for-age z-scores (WAZ), height-for-age z-scores (HAZ), weight-for-height z-scores (WHZ), and BMI-for-age z-scores BAZ), at baseline and after 6 months of intervention, in Nigerian toddlers with malnutrition, provided with a fortified dairy-based drink in daily amounts of 200, 400 or 600 ml: **(A)** WAZ, **(B)** HAZ, **(C)** WHZ, and **(D)** BAZ. The difference between baseline and endline was tested with a paired *t*-test per study group. *p < 0.05, **p < 0.01, ***p < 0.001, ****p < 0.0001.

Table 5 shows the estimated means of the anthropometric outcomes after the intervention. The three study groups all show an increase in weight and height during the study. Following the intervention, weight and height showed a (dose-response) trend to differ between the three study groups (*p* = 0.081 and *p* = 0.062, respectively). BMI, head-circumference and MUAC improved in all groups compared to baseline, but no differences were found between groups. Also, for the Z-scores, no differences were found between the groups. Only the WAZ showed a (dose-response) trend to differ between groups (*p* = 0.079). Within groups WAZ-, WHZ-, and BAZ scores significantly improved during the intervention (Figure 3). With regard to HAZ, only the 600 ml group improved from baseline to endline (*p* < 0.0001). When growth parameters were plotted in the WHO growth curves (43): (1) the absolute weights gradually moved from the 3^rd^ to the 15^th^ percentile line (Supplementary Figures 1A,B); (2) length or height followed more or less the 3^rd^ percentile line (Supplementary Figures 1C,D), at least for boys, whereas older girls (24–29 months) tended to improve their length towards the 15^th^ percentile; (3) BMI went from the 15^th^ percentile towards median values, in particular for the girls in the 600 ml group (Supplementary Figures 1E,F); and (4) head circumference followed the curve in between the 15^th^ percentile and median (Supplementary Figures 1G,H).

**Table 5:** Endline anthropometric data of the modified PP population of Nigerian toddlers with malnutrition who were provided with a fortified dairy-based drink in daily amounts of 200, 400 or 600 ml, during 6 months (data presented as means ± SE).

	**200 ml** **(***n*** = 32)**	**400 ml** **(***n*** = 36)**	**600 ml** **(***n*** = 31)**	***p*** **-value^a^**
Weight (kg)	10.36 ± 0.14	10.49 ± 0.13	10.79 ± 0.14	0.081
Height (cm)	82.39 ± 0.27	82.62 ± 0.25	83.29 ± 0.27	0.062
BMI (kg/m^2^)	15.16 ± 0.17	15.34 ± 0.16	15.60 ± 0.18	0.203
Head circumference (cm)	47.42 ± 0.17	47.69 ± 0.16	47.15 ± 0.17	0.071
Mid-upper arm circumference (MUAC) (cm)	14.32 ± 0.18	14.61 ± 0.17	14.56 ± 0.18	0.455
Weight-for Age z-score (WAZ)	−1.37 ± 0.10	−1.29 ± 0.10	−1.05 ± 0.11	0.079
Height-for-age z-score (HAZ)	−1.70 ± 0.09	−1.65 ± 0.08	−1.53 ± 0.09	0.395
Weight-for height z-score (WHZ)	−0.70 ± 0.13	−0.60 ± 0.12	−0.35 ± 0.13	0.150
BMI-for-age z-score (BAZ)	−0.48 ± 0.14	−0.37 ± 0.13	−0.12 ± 0.14	0.161
MUAC-z-score	−0.77 ± 0.15	−0.50 ± 0.14	−0.46 ± 0.15	0.278

a *The p-value represents the outcome of the Generalised Linear Model with the study arm as a factor and the corresponding baseline value for the outcome as a covariate*.

Average MUAC outcomes at base- and end-line, in combination with reference values, are presented in the Supplementary Materials (Supplementary Table 4). At baseline, the average calculated Z-scores for the 200-, 400- and 600 ml groups were −1.12, −0.98 and −0.78 SD, which are slightly better than Z-scores for weight and height (Table 2). At endline, the calculated Z-scores improved to −0.79, −0.52 and −0.39 SD, but groups were not different from each other.

### Cognition

Twenty-six children in the modified-PP population did not perform the Bayles-III Screening Test, due to no show or lack of cooperation of children. Seventy-three children at baseline and 72 at endline had data available for at least one sub-score of the test. Sixty-six subjects completed all subtests. Most missing values (*n* = 6) for subtests were seen in the 400 ml group at endline. Only 1 child in the 600 ml group did not perform a subtest at baseline. No missing values were seen in the 200 ml group. The reason for not completing all tests was a lack of concentration or willingness during the test. The absolute score values are presented in Supplementary Table 5. As indicated by the positive delta-values, all study groups improved their average scores for the different subtests. No differences were found between the study groups. Groups did not differ in the per cent of children per classification per development category after the intervention for any of the subtests (Supplementary Table 6). The only change observed within study groups was a decline in the percentage of competent children for receptive language (*p* = 0.03, *p* = 0.01, and *p* = 0.01 for the 200, 400, and 600 ml groups respectively).

## Discussion

In the present study, 200, 400, or 600 ml of a multi-nutrient fortified dairy-based drink was provided daily for 6 months to malnourished and anaemic Nigerian toddlers. The intake of 600 ml improved selenium (*p* < 0.05), vitamin A (*p* < 0.01), and vitamin D3 (*p* < 0.0001) status and consequently reduced the percentages of children who were deficient or had a sub-normal status for these micronutrients. For vitamin D, these effects were also seen in the 400 ml group. Groups did not differ in growth parameters, however, a trend towards differences between groups for height (*p* = 0.062) and weight (*p* = 0.081), suggests a dose-response effect. No differences were seen between interventions on cognitive subscores of the Bayley-III Screening Test.

### Effect of Multi-Fortified Dairy-Based Drink on Micronutrient Status

The present study shows that at baseline most children were not deficient in any of the nutrients studied. For vitamin B12 and folate this is in accordance with limited literature available for Northern Nigeria (23), but it is not in line with the reported low folate intake in the Ibadan region (24). The prevalence of vitamin D deficiency was 16.7% when <50 nmol/L was taken as a cut-off value, however, this cut-off is mainly supportive for skeletal metabolism (48, 57). For extra-skeletal activities, e.g., the immune system, a cut-off value of at least 75 nmol/L 25OHD is suggested (47, 48, 58–60). Vitamin D status is importantly determined by cutaneous synthesis following exposure to sunlight. For a pigmented skin, 1 to 1.5 h of sunlight on 25% of unprotected skin should be enough to ensure an sufficient vitamin D synthesis (61–63). However, the present study shows that close to 70% of the study population, with sufficient possibilities to be exposed to the sun in the absence of seasonality in cutaneous vitamin D synthesis (63), had <75 nmol/l 25OHD at baseline. The study shows that oral vitamin D intake resulted in an improvement of vitamin D status in all study groups. Based on the improvement in the 600 ml group from 67.0 nmol/L 25OHD at baseline to 78 nmol/L after 6 months, the response factor appears to be high (1.8 nmol/l per μg oral vitamin D) as might be expected in malnourished children (56). In healthy European children aged 1–3 years, receiving 8.5 μg additional vitamin D daily (64), and in Australian and New Zealand 1-y-old participants receiving 1.4 μg additional vitamin D daily (65), the response factor was around 1.1. Vitamin A deficiency (VAD) in Nigerian children under 5 years of age was earlier reported to be about 29.5% (66) despite the mandatory fortification of vegetable oil, wheat flour and sugar with vitamin A (67, 68). Repeated malaria infections could be a plausible factor as these infection have been associated with reduced vitamin A status in children (69). Baseline prevalence in the present study (35.5%) indicates that vitamin A deficiency has not improved. Restoring vitamin A, however, has shown to reduce all-cause and diarrhoea specific mortality in children under 5 years of age quite significantly (70). The reduction in the vitamin A deficiency as seen in the 400 ml and 600 ml groups from 32.3 to 23.5% and from 40.0 to 16.7%, respectively, is important and shows that daily supplementation with relatively low doses of vitamin A (255–383 μg-RE) might be very supportive for general health.

With regard to the minerals, plasma selenium concentrations in the present study show that 17.9% of children at baseline were selenium insufficient (<0.8 μmol/L), which is higher than estimated (1–10%) based on selenium intake (31). The prevalence of selenium deficiency decreased to 6.7%, 2.9% and 6.5% in the 200-, 400-, and 600-ml group respectively, at the end of the study. Plasma concentrations of 0.9 to 1.3 μmol/L (70 to 100 μg/L) are proposed to reflect selenium adequacy (71), whereas maximal platelet glutathione peroxidase activity is achieved at a plasma concentration of about 1.25–1.45 μmol/L (100–115 μg/L) (72). This might indicate that the currently used cut-off value for children is too low. An important role of selenium is being part of selenocysteine in the catalytic centre of enzymes protecting the thyroid from H_2_O_2_ which is released when iodine is used in the synthesis of thyroid hormones (33). A higher selenium status therefore would be very useful in the toddlers of the present study to cope with the relatively high iodine intake. Urinary median iodine excretion was 309 μg/L (range: 22–5622) at baseline, indicating an excessive iodine intake by these children. The high intake may be a result of the iodine-salt-fortification program, recommending 50 mg iodine fortification per kg of salt (10). The WHO recommends <5 g salt intake per day for adults (51), which is, based on energy requirements, and about 3 g for children 3 years of age. This amount provides 150 μg of iodine per day to toddlers whereas the recommendation is 90 μg/day (73), assuming that all salt is consumed as discretionary salt and not from processed foods. Zinc is important in stimulating growth in length and weight, with a stronger effect after 2 years of age (25). However, plasma zinc concentrations may be normal when it is already limiting growth (a so-called “type 2 nutrient”). When under zinc limiting conditions, more energy is provided to stimulate growth, zinc availability will be stressed which may result in a plasma zinc decrease (57). According to the National Food Consumption Survey 2003, 20% of children aged <5 years were found to be zinc deficient in Nigeria (28), which is in agreement with the present study (average deficiency of 17.2% at baseline). Although plasma zinc responds to supplementation, as shown in children supplemented with 7 or 10 mg/day (74), the present study did not show this effect (supplementing 1, 2 or 3 mg/day). For the latter finding, it is hypothesised that the low levels of zinc provided are not enough to compensate for the zinc requirement associated with stimulated growth by the dairy-based product, while for zinc adequate children no effect on zinc plasma levels might be seen due to a plateauing effect when zinc intakes are higher than the requirements, as suggested in healthy men (74).

### Effect of Multi-Fortified Dairy-Based Drink on Physical Growth

In developing countries, milk intake is associated with linear growth, in which at least the high-quality protein and bone-friendly components such as calcium play a role (41, 75, 76). Although protein intake might not be limited in many Nigerian children, the protein quality might not be optimal (41). The present study suggests that an increasing amount of daily multi-fortified dairy-based drink could be beneficial for linear growth. Children consuming 600 ml daily were 0.9 cm and 0.67 cm taller than those from the 200 and 400 ml groups, respectively. The negative anthropometric Z-scores at baseline improved during the intervention period in all groups. A possible effect of supplementation on height in the present study is consistent with a study conducted in 1002 preschool-age children (1–5 years) from the National Health and Nutrition Examination Survey (NHANES). This study showed that children who drank milk daily were taller (1.0 cm; *p* <0.02) than those with less frequent intake (76). A prospective cohort study among premenarchal girls who drank >3 servings per day of milk grew 0.28 cm more the following year than girls consuming <1 serving per day. Of the foods and nutrients studied, dairy protein had the strongest association with linear growth while non-dairy animal protein and vegetable protein were never significant, nor were non-dairy animal fat and vegetable fat (77). It is suggested that milk components stimulate IGF-I concentrations and, thereby, growth (75, 76, 78–84).

### Cognition

With the use of the Screening Test of the Bayley-III, no effects of the intervention were found between or within groups. The percentage of children at risk (for any of the subtests) varied considerably from 0 to 38.1%. For receptive language (how the child understands language) a decline in classification was seen in all three study groups. Important factors associated with poor development are non-stimulating home environments, a limited role of the father in child-raising, or low social-economic status of the family (associated with poor nutrition and stunting) (3, 85, 86). The reported decline in receptive language might be a consequence of the low social and economic conditions (80% of the households in the studied population) in combination with undernutrition (all were malnourished and anaemic children) (3). Also, the fact that many children are exposed to more than one language at home (more than 250 native languages spoken in Nigeria) might have played a role, though English is the official language. The improvements in nutritional status and anthropometry apparently could not prevent a decrease in receptive language development, at least not within the 6-month study period.

### Strengths and Limitations

Although it was not the goal of the study, the absence of a placebo group is a limitation, preventing the insight into the effect of the lowest amount of product intake. Therefore, in a future study, it would be of interest to follow up a non-supplemented control group as well. In this study we had an intervention period of 6 months; while this was sufficient to see improvements in micronutrient status, a longer study period appears to be necessary to see real improvements in cognition. For a future study, an intervention duration of 12 months would be advised. With regard to the cognitive data, the screening version of the Bayley-III tool does not allow to make diagnostic interpretations. Besides, the test had quite a lot of dropouts and faced a lack of concentration and willingness to do or complete the test. Therefore, it is difficult to draw any firm conclusions from this dataset. The absence of food intake data in this manuscript makes it difficult to attribute the improvements observed to the fortified dairy-based drink only. Since children with medically diagnosed allergies, not having anaemia, intolerances to milk or milk ingredients were excluded, and since children were recruited from a poor environment, study results cannot directly be generalised to children with different characteristics. A strength of this study is the food-based dose-response approach, showing that an intake of 200 ml of the study product already can have beneficial effects on growth (improved z-scores).

## Conclusions

This study showed that daily consumption of a multi-nutrient fortified dairy-based drink by toddlers improved their nutritional status of vitamin A (600 ml), vitamin D (400 and 600 ml) and selenium (600 ml). The latter can be important to cope with a possible excessive intake of iodine by Nigerian children as indicated in the present study. The effect on zinc status is not clear due to the absence of a placebo, the amount provided and/or study duration. With regard to growth, z-scores of weights, height, and BMI improved as compared to baseline, but no difference was seen between groups. No effects were seen on cognitive development. For anthropometry and cognition, a longer study duration might be necessary. The most beneficial daily amount of the fortified diary-based drink appears to be 600 ml.

## Data Availability Statement

The original contributions presented in the study are included in the article/Supplementary Material, further inquiries can be directed to the corresponding author/s.

## Ethics Statement

The studies involving human participants were reviewed and approved by the Ethics Committee of Lagos State University Teaching Hospital and the Lagos State Government (LREC/10/06/829). The trial was registered at ClinicalTrials.gov: NCT03411590. Written informed consent to participate in this study was provided by the participants' legal guardian/next of kin.

## Author Contributions

AJO, AS, and IOS designed the study. IOS, KAO, YA, and AJO conducted the study. JHJH, AJO, and AM-B analyzed the data. AM-B, FS, and AS supervised the study. All authors contributed to writing, editing and agreed to the published version of the manuscript.

## Funding

This study was sponsored by FrieslandCampina, Amersfoort, Netherlands.

## Conflict of Interest

JHJH and AS are employees of FrieslandCampina. At the time the study was conducted AJO was employed at FrieslandCampina WAMCO, Nigeria. The remaining authors declare that the research was conducted in the absence of any commercial or financial relationships that could be construed as a potential conflict of interest.

## Publisher's Note

All claims expressed in this article are solely those of the authors and do not necessarily represent those of their affiliated organizations, or those of the publisher, the editors and the reviewers. Any product that may be evaluated in this article, or claim that may be made by its manufacturer, is not guaranteed or endorsed by the publisher.
