# Supplementary material for: Effect of a Fortified Dairy-Based Drink on Micronutrient Status, Growth, and Cognitive Development of Nigerian Toddlers- A Dose-Response Study
Source: Front Nutr. 2022 Apr 27;9:864856. doi: 10.3389/fnut.2022.864856 (PMC9097016; doi:10.3389/fnut.2022.864856)
Supplement: Supplementary file 1 [file Table_1.DOCX]

Effect of a fortified dairy-based drink on micronutrient status, growth, and cognitive development of Nigerian toddlers- A dose-response study

Supplementary Material

**Supplemental Table 1**: List of Ingredients

| List of ingredients |
| --- |
| Skimmed milk, Glucose syrup solids, Vegetable oils, Palm oil, Canola oil (low erucic acid type), Palm kernel oil, Sunflower oil, Saccharose, Fish oil, Sodium L-ascorbate, Emulsifier (Lecithin), Lactose, Taurine, Meso-inositol, Choline chloride, Ferrous sulphate, Vanilla flavour, DL M-tocopheryl acetate, Zinc sulphate, L-Ascorbyl palmitate, Nicotinamide, Manganese sulphate, Calcium D-pantothenate, Thiamin hydrochloride, Cupric sulphate, Retinyl-acetate, Pyridoxine hydrochloride, ß-carotene, Folic acid, Potassium iodide, Phytomenadione, D-Biotin, Cholecalciferol, Sodium selenite. |

**Supplemental Table 2:** Bayley-III Screening Test: an overview of the subtest scores used for classification

|  |  | **Total Raw Score** | | |
| --- | --- | --- | --- | --- |
| **Age category** | **Subtests** | **At risk** | **Emerging** | **Competent** |
| Ages 12 months 16 days – 18 months 15 days | Cognitive | 0-13 | 14-16 | 17-33 |
|  | Receptive Communication | 0-9 | 10-11 | 12-24 |
|  | Expressive Communication | 0-9 | 10-12 | 13-24 |
|  | Fine Motor | 0-10 | 11-13 | 14-27 |
|  | Gross Motor | 0-12 | 13-16 | 17-28 |
| Ages 18 months 16 days – 24 months 15 days | Cognitive | 0-16 | 17-20 | 21-33 |
|  | Receptive Communication | 0-11 | 12-15 | 16-24 |
|  | Expressive Communication | 0-11 | 12-15 | 16-24 |
|  | Fine Motor | 0-11 | 12-16 | 17-27 |
|  | Gross Motor | 0-16 | 17-18 | 19-28 |
| Ages 24 months 16 days – 30 months 15 days | Cognitive | 0-20 | 21-24 | 25-33 |
|  | Receptive Communication | 0-12 | 13-18 | 19-24 |
|  | Expressive Communication | 0-12 | 13-18 | 19-24 |
|  | Fine Motor | 0-14 | 15-18 | 19-27 |
|  | Gross Motor | 0-18 | 19-21 | 22-28 |
| Ages 30 months 16 days – 36 months 15 days | Cognitive | 0-22 | 23-27 | 28-33 |
|  | Receptive Communication | 0-12 | 13-20 | 21-24 |
|  | Expressive Communication | 0-14 | 15-20 | 21-24 |
|  | Fine Motor | 0-17 | 18-22 | 23-27 |
|  | Gross Motor | 0-18 | 19-23 | 24-28 |
| Ages 36 months 16 days – 42 months 15 days | Cognitive | 0-24 | 25-31 | 32-33 |
|  | Receptive Communication | 0-14 | 15-22 | 23-24 |
|  | Expressive Communication | 0-15 | 16-23 | 24 |
|  | Fine Motor | 0-17 | 18-25 | 26-27 |
|  | Gross Motor | 0-20 | 21-26 | 27-28 |

**Supplemental Table S3:** Baseline characteristics of ITT and modified PP populations of malnourished Nigerian toddlers provided with a fortified dairy-based drink in daily amounts of 200, 400 or 600 ml, during 6 months.

|  | **ITT** | **Modified PP** |
| --- | --- | --- |
| **N** | 165 | 99 |
| **Age** **(months)** | 20.2 ± 6.3 | 20.0 ± 6.2 |
| **Gender** **(boys/girls) (%)** | 44.8 / 55.2 | 47.5 / 52.5 |
| **Social class (upper/middle/lower) (%)** | 0.6 / 18.4 / 81.0 | 1.0 / 19.6 / 79.4 |
| **Religion (Muslim/Christian) (%)** | 71.0 / 29.0 | 69.1 / 30.9 |
| **Weight (kg)** | 8.9 ± 1.2 | 8.9 ± 1.2 |
| **Height (cm)** | 77.5 ± 4.9 | 77.6 ± 4.9 |
| **Head circumference (cm)** | 46.6 ± 1.6 | 46.6 ± 1.6 |
| **Waist circumference (cm)** | 44.8 ± 3.1 | 44.8 ± 3.2 |
| **Mid-upper arm circumference (cm)** | 13.7 ± 0.9 | 13.8 ± 0.9 |
| **Weight for age Z- score** | -1.80 ± 0.56 | -1.76 ± 0.57 |
| **Height for age Z- score** | -1.80 ± 0.65 | -1.78 ± 0.61 |
| **Weight for height Z-score** | -1.24 ± 0.78 | -1.20 ± 0.75 |
| **BMI for age Z-score** | -0.95 ± 0.78 | -0.95 ± 0.79 |
| **Iodine** | 311.0, 434.0 | 297.2, 424.4 |
| **Selenium** | 0.9, 0.3 | 0.9, 0.2 |
| **Zinc** | 11.4, 2.6 | 11.3, 2.7 |
| **Vitamin A (umol/L)** | 0.8, 0.4 | 0.8, 0.4 |
| **Vitamin B12 (pmol/L)** | 579.0, 341 | 602.5, 324 |
| **Folate (nmol/L)** | 19.3, 12.9 | 21.0 ± 9.1 |
| **Vitamin D3 (nmol/L)** | 66.0, 25 | 67.0, 23 |

*Data are presented as mean ± SD or median, IQR.*


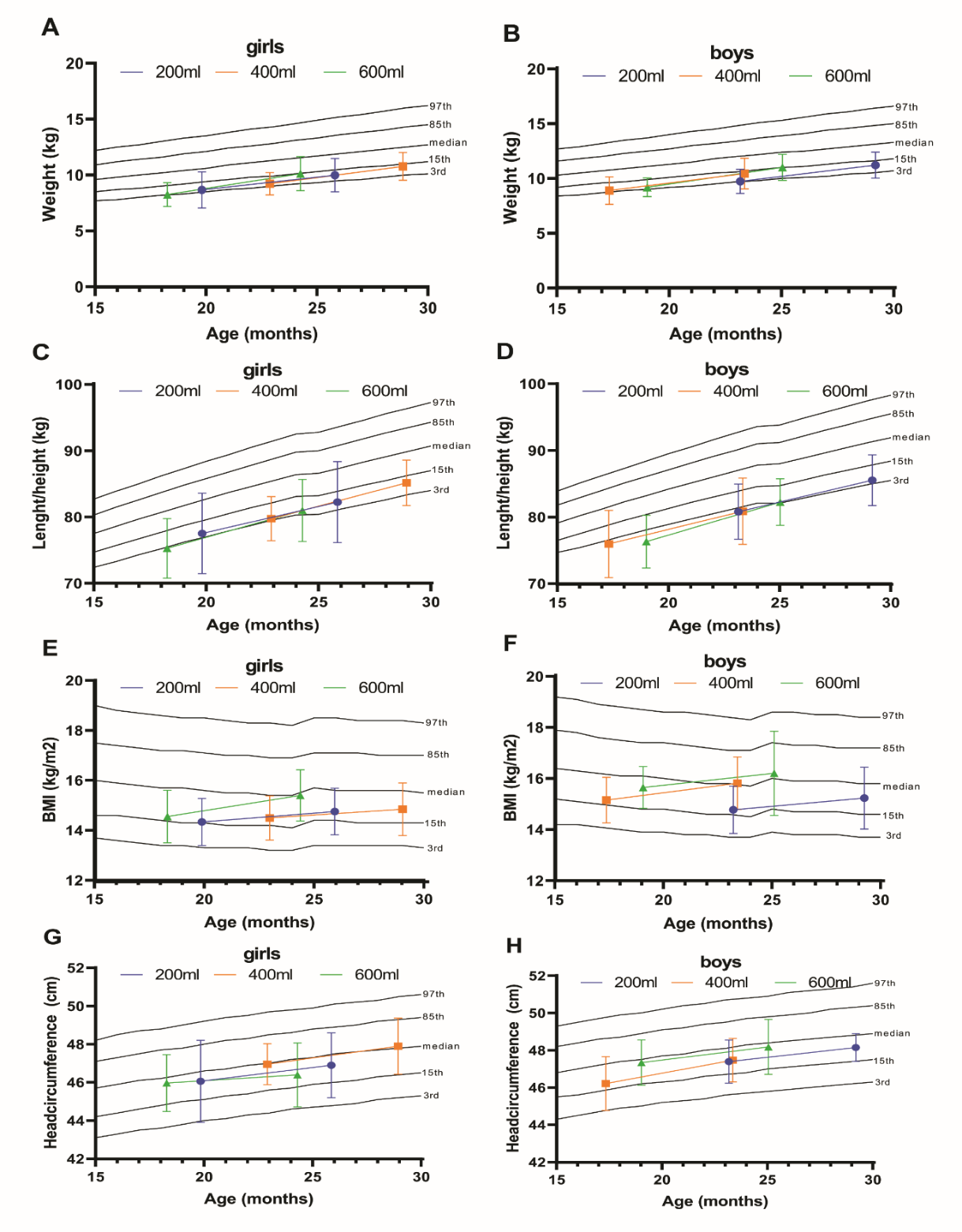


**Figure S1**: **A)** Weight of girls, **B)** weight of boys, **C)** length/height of girls, **D)** length/height of boys, **E)** BMI of girls, **F)** BMI of boys, **G)** head circumference of girls and **F)** head circumference of boys of the different intervention groups (200, 400 and 600 ml) in the modified PP population of malnourished Nigerian toddlers, at the start and after the intervention period. Data are plotted in WHO growth curves presenting with the 3^rd^, 15^th^, median, 85^th^ and 97^th^ percentiles of the growth curves.

**Supplemental Table S4:** MUAC age-reference values for boys and girls aged 6-59 months (1), and mean outcomes (at base- and end-line) in Nigerian toddles provided with a fortified dairy-based drink in daily amounts of 200, 400 or 600 ml, during 6 months.

|  |  | **Current study** | | **MUAC reference values and Z-scores** (1) | | | | | | | |
| --- | --- | --- | --- | --- | --- | --- | --- | --- | --- | --- | --- |
| **Product group** |  | **Mean age (months)** | **Average MUAC (cm) (min-max)** | **-4SD** | **-3SD** | **-2SD** | **-1SD** | **MEAN** | **1SD** | **2SD** | **3SD** |
| **Ba*s*eline** | |  |  |  |  |  |  |  |  |  |  |
| **200ml** |  | 21 | 13.7 (12.0-15.5) | 10.8 | 12.1 | 13.4 | 14.7 | 16 | 17.3 | 18.7 | 20 |
| **400ml** |  | 20 | 13.7 (12.3-15.1) | 10.7 | 12.1 | 13.4 | 14.7 | 16 | 17.3 | 18.6 | 19.9 |
| **600ml** |  | 18 | 13.8 (12.5-15.7) | 10.7 | 12 | 13.3 | 14.6 | 15.9 | 17.2 | 18.5 | 19.8 |
| **Endline** | |  |  |  |  |  |  |  |  |  |  |
| **200ml** |  | 27 | 14.3 (12.5-17.0) | 10.8 | 12.2 | 13.5 | 14.9 | 16.2 | 17.6 | 18.9 | 20.3 |
| **400ml** |  | 26 | 14.6 (13.0-18.8) | 10.8 | 12.2 | 13.5 | 14.9 | 16.2 | 17.5 | 18.9 | 20.3 |
| **600ml** |  | 24 | 14.6 (12.5-16.5) | 10.8 | 12.1 | 13.5 | 14.8 | 16.1 | 17.5 | 18.8 | 20.2 |

**Supplemental Table S5:** Average absolute values of the subtest scores of the Bayley Screening III test at baseline and after the intervention in the modified PP population of Nigerian toddlers provided with a fortified dairy-based drink in daily amounts of 200, 400 or 600 ml, during 6 months. (mean ± SD or median, IQR).

|  | | **200 ml** | | **400 ml** | |  | | **p-value*** |
| --- | --- | --- | --- | --- | --- | --- | --- | --- |
| **Subtest** | | **baseline** | **endline** | **baseline** | **endline** | **Baseline** | **endline** |  |
| **Cognition** | Absolute average (mean±SD) | 21.0 ± 2.9  (n=23) | 23.4 ± 3.0 (n=23) | 20.3 ± 3.4 (n=29) | 23.0 ± 2.7 (n=24) | 18.6 ± 2.8 (n=21) | 21.8 ± 2.3 (n=21) |  |
|  | Delta (mean±SD) | 2.8 ± 2.6 (n=23) | | 3.2 ± 2.9 (n=24) | | 3.2 ± 2.1 (n=21) | | 0.851 |
| **Receptive language** | Absolute average (mean±SD or median,IQR) | 14.0, 3 (n=23) | 16.2 ± 2.6 (n=23) | 14.0, 2 (n=29) | 14.9 ± 2.9 (n=24) | 13.0, 2 (n=21) | 14.5 ± 1.9 (n=21) |  |
|  | Delta (mean±SD) | 1.7 ± 2.0 (n=23) | | 0.9 ± 2.4 (n=24) | | 1.4 ± 2.1 (n=21) | | 0.424 |
| **Expressive language** | Absolute average (mean±SD or median,IQR) | 14.0, 3 (n=23) | 15.7 ± 3.4 (n=23) | 12.0, 5 (n=29) | 15.5 ± 4.1 (n=28) | 13.0, 3 (n=21) | 13.3 ± 2.7 (n=21) |  |
|  | Delta (mean±SD) | 1.4 ± 2.6 (n=23) | | 2.4 ± 3.5 (n=28) | | 0.7 ± 2.1 (n=21) | | 0.116 |
| **Fine motor skills** | Absolute average (median,IQR) | 13.0, 4 (n=23) | 16.0, 8 (n=23) | 13.0, 3 (n=29) | 15.0, 5 (n=28) | 12.0, 3 (n=20) | 14.0, 3 (n=21) |  |
|  | Delta (mean±SD) | 2.3 ± 2.9 (n=23) | | 2.4 ± 3.4 (n=28) | | 1.8 ± 2.2 (n=20) | | 0.744 |
| **Gross motor skills** | Absolute average (median,IQR) | 19.0, 5 (n=23) | 23.0, 5 (n=23) | 19.0, 2 (n=24) | 20.0, 5 (n=28) | 18.0, 5 (n=21) | 20.0, 3 (n=21) |  |
|  | Delta (mean±SD) | 2.3 ± 2.3 (n=23) | | 2.5 ± 3.3 (n=24) | | 2.8 ± 3.5 (n=21) | | 0.873 |

**The p-value represents the outcome of a one-way ANOVA which was used to compare the delta-values of the subtests of the different study groups*

**Supplemental Table S6:** Percentages of classification groups of the subtest scores of the Bayley Screening III test at baseline and after the intervention in the modified PP population of Nigerian toddlers provided with a fortified dairy-based drink in daily amounts of 200, 400 or 600 ml, during 6 months.

|  | | **200 ml** | | **400 ml** | | **600 ml** | | **p-value*** |
| --- | --- | --- | --- | --- | --- | --- | --- | --- |
| **Subtest** | | **baseline** | **endline** | **baseline** | **endline** | **baseline** | **Endline** |  |
| **Cognition** | at risk (%) | 17.4 | 13.0 | 17.2 | 12.5 | 28.6 | 28.6 | 0.347 |
|  | emergent (%) | 34.8 | 34.8 | 20.7 | 37.5 | 23.8 | 14.3 |  |
|  | competent (%) | 47.8 | 52.2 | 62.1 | 50.0 | 47.6 | 57.1 |  |
|  | **p-value^** | 1.00 | | 0.38 | | 0.78 | |  |
| **Receptive language** | at risk (%) | 0.0 | 13.0 | 3.4 | 20.8 | 4.8 | 4.8 | 0.452 |
|  | emergent (%) | 65.2 | 78.3 | 44.8 | 62.5 | 42.9 | 85.7 |  |
|  | competent (%) | 34.8 | 8.7 | 51.7 | 16.7 | 52.4 | 9.5 |  |
|  | **p-value^** | **0.03** | | **0.01** | | **0.01** | |  |
| **Expressive language** | at risk (%) | 13.0 | 21.7 | 24.1 | 28.6 | 19.0 | 38.1 | 0.612 |
|  | emergent (%) | 65.2 | 60.9 | 48.3 | 42.9 | 47.6 | 42.9 |  |
|  | competent (%) | 21.7 | 17.4 | 27.6 | 28.6 | 33.3 | 19.0 |  |
|  | **p-value^** | 0.83 | | 0.94 | | 0.39 | |  |
| **Fine motor skills** | at risk (%) | 8.7 | 17.4 | 13.8 | 35.7 | 35.0 | 33.3 | 0.292 |
|  | emergent (%) | 69.6 | 52.2 | 62.1 | 39.3 | 45.0 | 57.1 |  |
|  | competent (%) | 21.7 | 30.4 | 24.1 | 25.0 | 20.0 | 9.5 |  |
|  | **p-value^** | 0.52 | | 0.12 | | 0.58 | |  |
| **Gross motor skills** | at risk (%) | 8.7 | 8.7 | 12.5 | 17.9 | 14.3 | 9.5 | 0.684 |
|  | emergent (%) | 13.0 | 17.4 | 16.7 | 28.6 | 42.9 | 23.8 |  |
|  | competent (%) | 78.3 | 73.9 | 70.8 | 53.6 | 42.9 | 66.7 |  |
|  | **p-value^** | 1.00 | | 0.49 | | 0.32 | |  |

**these p-values represent the outcome of a Fisher’s Exact test in which the prevalences of the classification groups were tested between study groups at endline, ^these p-values represent the outcome of the Fisher’s exact test that was used to compare the baseline and endline percentages within study groups.*

**Reference**

1. De Onis M, Yip R, Mei Z. The development of MUAC-for-age reference data recommended by a WHO Expert Committee. Bull World Health Organ. 1997;75(1):11–8.
